# Supplementary material for: The interactome of intact mitochondria by cross-linking mass spectrometry provides evidence for coexisting respiratory supercomplexes
Source: Mol Cell Proteomics. 2017 Dec 8;17(2):216–32. doi: 10.1074/mcp.RA117.000470 (PMC5795388; doi:10.1074/mcp.RA117.000470)
Supplement: Supplemental Data [file supp_RA117.000470_133922_0_supp_23978_fzffwf.zip › spectra_annotation/mito_DR_spectra_annotation/196-1-11-1-4-1.pdf]

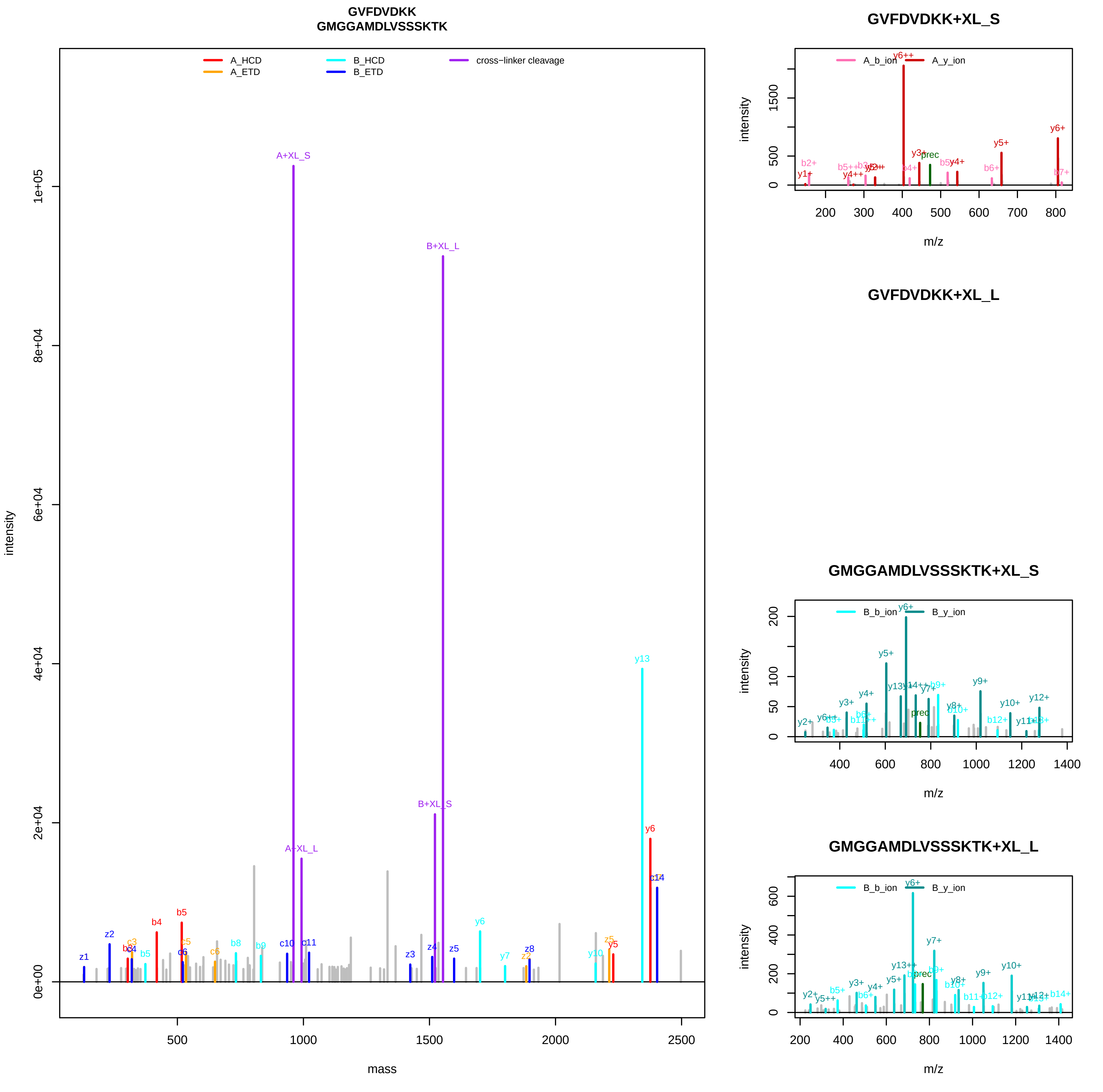

GVFDVDKK
GMGGAMDLVSSSKTK
A_HCD
A_ETD
B_HCD
B_ETD
cross−linker cleavage
A+XL_S
B+XL_L
B+XL_S
A+XL_L
y13
y6
c14
y5
z5
y10
z8
z2
y7
y6
z5
z4
z3
c11
c10
b9
b8
c6
c5
c3
c4
b5
b4
z2
z1
b5
mass
intensity
GVFDVDKK+XL_S
A_b_ion
A_y_ion
y6++
y6+
y5+
y4+
y3+
prec
b2+
y1+
b6+
b7+
m/z
intensity
GVFDVDKK+XL_L
GMGGAMDLVSSSKTK+XL_S
B_b_ion
B_y_ion
y6+
y5+
y4+
y3+
y9+
y10+
y12+
y7+
b9+
prec
y8+
b10+
b12+
y2+
m/z
intensity
GMGGAMDLVSSSKTK+XL_L
y7+
y10+
y9+
y8+
b9+
prec
b5+
y2+
b14+
m/z
intensity
